# Corrosion Behavior of HVAF-Sprayed WC-10Co-4Cr Coatings in H_2_SO_4_ and HNO_3_ Environments

**DOI:** 10.3390/ma19112343

**Published:** 2026-06-01

**Authors:** Yanli Chen, Weicai Wan, Mengxia Liang, Shengyun Xiao, Wei Liu, Jiupeng Song, Kunyang Fan

**Affiliations:** 1 Key Laboratory of Materials and Surface Technology (Ministry of Education), School of Materials Science and Engineering, Xihua University, Chengdu 610039, China; 19150117196@163.com (Y.C.); cronopio2025@163.com (S.X.); liuwei99072022@163.com (W.L.); sjp@mail.xhu.edu.cn (J.S.); 2 School of Mechanical Engineering, Chengdu University, Chengdu 610106, China; fankunyang@cdu.edu.cn

**Keywords:** HVAF, WC-10Co-4Cr coating, grain size, corrosion behavior

## Abstract

Three WC-10Co-4Cr coatings with different WC grain sizes were prepared by high-velocity air-fuel (HVAF) spraying. The corrosion behaviors were systematically evaluated in 0.2 mol/L H_2_SO_4_ and 0.4 mol/L HNO_3_ solutions through immersion tests and electrochemical measurements. The results reveal that WC grain size governs coating microstructural integrity, mechanical properties, and corrosion resistance. Among the three coatings, the medium-grained (MG) coating exhibits an optimized balance between compact microstructure, high microhardness, superior fracture toughness, and the best corrosion resistance in both acidic environments. The coarse-grained (CG) coating exhibits the worst corrosion resistance owing to its wide grain boundaries and high porosity, while the fine-grained (FG) coating is similarly compromised by slightly higher porosity and residual stress-induced microcrack networks that facilitate electrolyte penetration. The corrosion proceeds via preferential dissolution of Co in the CoCr binder phase driven by micro-galvanic coupling with WC, followed by WC particle detachment and pit formation. In a 0.4 mol/L HNO_3_ solution, the strong oxidizing nature accelerates both binder dissolution and direct WC oxidation.

## 1. Introduction

Tungsten carbide (WC)-based cermet coatings, particularly in the WC-Co and WC-Co-Cr systems, are among the most important protective coatings in surface engineering. This is primarily because the combination of a hard WC phase and a ductile metallic binder phase (e.g., Co or CoCr) provides an exceptional balance of high hardness, outstanding wear resistance, and favorable corrosion resistance. Consequently, they have been extensively applied in marine engineering, petrochemical sectors, energy equipment, and aerospace applications [1,2]. In recent years, high-velocity air-fuel (HVAF) spraying has garnered escalating attention as a competitive alternative for depositing WC-based hard coatings. Compared with conventional high-velocity oxygen-fuel (HVOF) spraying, HVAF utilizes lower-cost, higher-pressure compressed air as the combustion-supporting agent, thereby drastically reducing the processing cost. Concurrently, the lower flame temperature and oxygen-lean atmosphere inherent in HVAF spraying effectively suppress the decarburization and oxidation of WC, thus mitigating the porosity of the as-deposited coatings [3,4,5,6]. Consequently, HVAF-sprayed coatings generally exhibit superior wear and corrosion resistance, positioning HVAF as a promising technique for fabricating high-performance cemented carbide coatings [7]. For example, Owoseni et al. [8] recently revealed that HVAF-sprayed WC-CoCr coatings on aluminum alloy achieved a specific wear rate as low as 1.7 × 10^−8^ mm^3^/Nm, markedly superior to that of HVOF coatings (16.7 × 10^−8^ mm^3^/Nm). Lakshmi et al. have further substantiated the advantages of the HVAF process. Thin WC-10Co-4Cr coatings (≈100 µm) deposited by HVAF exhibit approximately 2–4 times higher corrosion resistance than conventional thick coatings (≈300 µm), and carbide size significantly influences the corrosion rate [9]. These results confirm the technological advantages of HVAF for fabricating high-performance WC-based coatings.

In contrast to conventional WC–Co cermet systems, WC–Co–Cr coatings have garnered widespread research attention due to their superior synergy of mechanical properties and corrosion resistance [10,11]. Prior investigations have validated the corrosion-enhancing effect of Cr addition: corrosion tests on WC–6Co, WC–11Co, and WC–16.5Co cermet coatings in sulfuric acid and simulated seawater environments revealed that Cr-modified WC–Co coatings possess markedly enhanced corrosion resistance relative to their Cr-free counterparts [2,12]. Further studies focusing on the corrosion behavior of WC–Co-based coatings under diverse pH conditions similarly demonstrated that Cr-free WC–Co coatings exhibit significantly higher corrosion rates than Cr-containing coatings [13,14,15]. Additionally, Zhou et al. [16,17,18] deposited WC–Co and WC–Co–Cr coatings onto 45 steel substrates and explored their corrosion mechanisms via electrochemical characterization, and the corresponding results indicated that the WC–Co–Cr coating possessed the maximum charge transfer resistance, confirming that Cr addition substantially boosts the coating’s corrosion resistance. This notable improvement is primarily ascribed to the formation of a CoCr alloy binder phase (via partial Cr dissolution), which exhibits far superior corrosion resistance compared to the pure Co binder phase [14,19]. Recently, Wang et al. [20] compared the tribocorrosion behaviors of WC-10Co-4Cr, WC-20Cr_3_C_2_-7Ni, and Cr_3_C_2_-25NiCr cermet coatings fabricated by HVAF thermal spraying and demonstrated that HVAF-sprayed WC-10Co-4Cr coatings exhibit the best tribocorrosion resistance owing to the higher hardness of WC and more uniform phase distribution. They also presented that a higher concentration of Cr in the coatings facilitates the development of a comprehensive and continuous Cr_2_O_3_ protective film, which exhibits enhanced shear properties, delivering more effective interfacial lubrication and thereby substantially reducing the friction coefficient. By introducing the Cr element in the form of Cr_2_(C,N) into the WC-Co cemented carbide system, He et al. [21] found that the tendency for preferential corrosion induced by Co pools can be effectively mitigated. The Cr element is oxidized during polarization to form a dense Cr_2_O_3_ passive film that acts together with other corrosion products to resist attack by the corrosive medium, thereby significantly enhancing the corrosion resistance. These results offer a deeper understanding of the Cr modification mechanism.

In general, grain refinement increases the total interfacial area and promotes the formation of galvanic couples at the material surface. Both factors are theoretically expected to compromise the corrosion resistance of metallic/ceramic composite systems. However, the limited published literature regarding the effect of WC grain size on the corrosion behavior of WC-Co cemented carbide presents inconsistent and inconclusive findings. Tomlinson et al. [22] reported that passive current density increased with grain size in acidic media. Su et al. further found that by introducing Cu and Ni into ultra-coarse grain WC-8Co cemented carbides to modify the binder phase, the corrosion resistance in acidic solution could be significantly enhanced [23]. In NaOH solution, Kellner et al. [24] observed that the corrosion resistance of WC–Co hardmetals increases with decreasing grain size. They indicated that the effect of grain size, which contributes to fcc Co phase stabilization, plays a dominant role in the corrosion behavior. Smaller grain WC–Co hardmetals are enriched in W and C in the Co binder, which leads to a higher amount of fcc Co in the binder, resulting in lower current densities upon polarization. Recently, Zhang et al. [25] observed a similar trend that the corrosion resistance of cemented carbides decreases with increasing WC grain size, with grain boundaries acting as preferential corrosion attack sites. However, Human et al. [26,27] observed that grain size had a negligible effect on the corrosion behavior of WC-based hardmetals in acidic solutions.

Although these recent studies have advanced our understanding, they have mostly focused on bulk cemented carbides prepared by conventional powder metallurgy rather than thermally sprayed coatings. In addition, owing to the substantial discrepancies in the materials and electrolytes across these investigations, a definitive interpretation of the underlying corrosion mechanisms of WC-10Co-4Cr coatings remains unelucidated at this stage. The effects of WC grain sizes on corrosion of WC-10Co-4Cr coatings, particularly in acidic media, remain unclear. This strongly restricts the wider application of WC-10Co-4Cr coatings.

In order to explore the service properties under typical industrial acidic conditions, WC-10Co-4Cr coatings with various WC grain sizes are fabricated via HVAF thermal spraying in this work, and their corrosion behaviors are systematically investigated in two distinct aggressive acidic environments: 0.2 mol/L H_2_SO_4_ and 0.4 mol/L HNO_3_. The selection of these two acidic media allows for a comparative analysis of corrosion mechanisms under distinct conditions—H_2_SO_4_ primarily induces binder phase dissolution (the dominant failure mode for WC-based coatings in acidic environments), whereas HNO_3_, as a strong oxidizing acid, promotes the formation of surface passive films such as WO_3_, enabling an assessment of the interplay between WC grain size and passivation behavior. By systematically correlating WC grain size with electrochemical responses in these two media, this work aims to elucidate the underlying corrosion mechanisms of HVAF-sprayed WC-based coatings and to provide fundamental guidance for the grain size design of high-performance cermet coatings tailored for aggressive acidic environments.

## 2. Materials and Methods

### 2.1. Materials and High-Velocity Air Fuel Spraying

**Figure 1:**
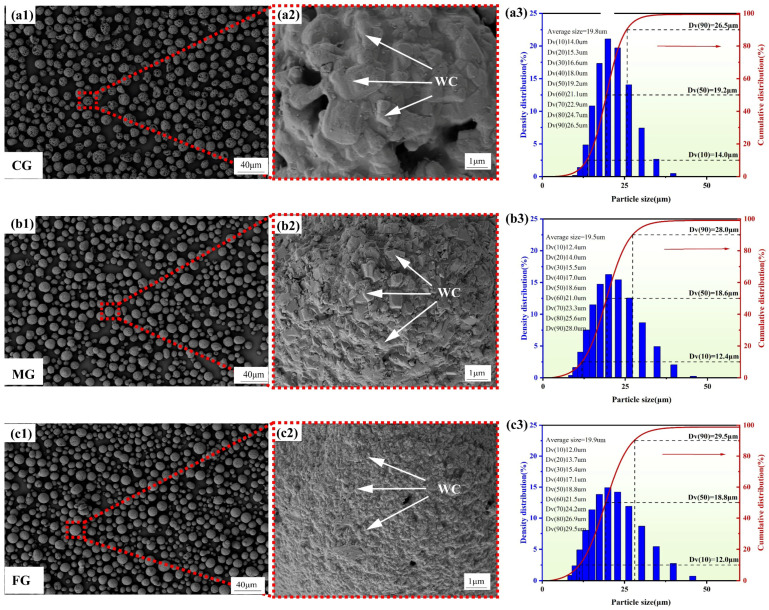
SEM morphologies of the WC-10Co-4Cr powders with various WC particle sizes and their spherical particle size distribution: CG (**a1**–**a3**); MG (**b1**–**b3**); FG (**c1**–**c3**).

Three types of commercial WC-10Co-4Cr (wt.%) powders with different WC particle sizes, manufactured by Luoyang Jinlu Carbide Tools Co., Ltd. (Luoyang, China), were utilized as HVAF feedstocks. Figure 1 presents the SEM morphology and spherical particle size distribution of the purchased sintered WC-10Co-4Cr powders. In Figure 1(a1–c1), three WC-10Co-4Cr powder types are spherical, and inter-particle agglomeration was not detected. Particularly, the enlarged images in Figure 1(a2–c2) show that the irregularly shaped WC particles are wrapped by the CoCr binder phase. The WC-10Co-4Cr powders with coarse, medium, and fine WC particles are designated as CG, MG, and FG, respectively. Their WC particle size ranges are 1.5–3 µm, 0.5–1.5 µm, and 0.2–0.5 µm, respectively. Overall, the three powder types have similar spherical particle size distribution (see Figure 1(a3–c3)), which is beneficial to excellent flowability during HVAF spray and can largely guarantee the uniform chemical distribution and microstructure of sprayed coatings. A total of 304 stainless steel (304 SS) plates of 15 mm thick (supplied by Taiyuan Iron and Steel Co., Ltd., Taiyuan, China) were employed as the substrate for HVAF spraying. Prior to the spraying procedure, sandblasting was performed using 200–400 mesh Al_2_O_3_ abrasive to remove surface oxide scale and roughen the substrate, with the primary objective of enhancing the coating–substrate bond strength.

The WC-10Co-4Cr coatings on 304 SS were prepared using an HVAF spraying system (Model M2, UNIQUECOAT Inc., Oilville, VA, USA). Industrial-grade propane (purity ≥ 99.5%) was used as fuel gas, with dry compressed air serving as the oxidizer, ensuring a controlled flame temperature and minimizing powder oxidation during spraying. Nitrogen was employed as an inert protective gas as well as a transmission medium of spraying powder from the feeder to the spray gun. The optimized HVAF spraying parameters are listed in Table 1.

### 2.2. Characterization

To examine the microstructure and chemical composition of the coating specimen prepared by HVAF, the sample was sectioned into small cubes with dimensions of 10 mm × 10 mm × 5 mm using wire electrical discharge machining. Subsequently, the samples were ground with diamond abrasive papers in the order of 120, 300, 600, 1200, and 2000 grits, followed by polishing with 1.5 μm diamond paste. After that, the sample surfaces were ultrasonically cleaned in anhydrous ethanol using an ultrasonic cleaner (JP-010T, Shenzhen Jiemeng Cleaning Equipment Co., Ltd., Shenzhen, China) and dried with a hair dryer. The coating microstructure was examined using a scanning electron microscope (SEM, S-360, ZEISS, Oberkochen, Germany), with elemental composition analyzed by an energy-dispersive X-ray spectrometer (EDS, Ultim Max, Oxford, UK). Phase composition was determined by X-ray diffraction (XRD, DX-2700BH, Dandong, China). Further, electron backscatter diffraction (EBSD, detector Symmetry S3 equipped on SEM) with a step size setting of 0.06 μm was used to characterize the grain and phase structures of coatings.

The density of the coatings was measured using a balance (BS224S, Sartorius, Beijing, China) with a sensitivity of 0.0001 g, following the Archimedean method at room temperature (23 °C).

The hardness of the coatings was measured using a Vickers hardness tester (HV-30, Laizhou Huaze Testing Instrument Co., Ltd., Laizhou, China) under a load of 30 kgf. The indentation fracture toughness K_IC_) of the cermet materials was calculated using the Vickers hardness according to Equation (1) [28].

The bond strength between the coating and the substrate was measured using a universal testing machine (WDW-50, Jinan Shijin Group Co., Ltd., Jinan, China), and the test procedure was carried out in accordance with the GB/T 8642-2025 [29] standard.

(1) $$K_{IC}=0.15\sqrt{{HV}_{30}/\sum_{i=1}^{4}L_{i}}$$

here, ${HV}_{30}$ represents the measured Vickers hardness (N/mm^2^), and $L_{i}$ is the length from the crack tip to the indentation (mm).

### 2.3. Corrosion Experiments

The corrosion behaviors of WC-10Co-4Cr coatings in 0.2 mol/L H_2_SO_4_ and 0.4 mol/L HNO_3_ solutions were evaluated. First, immersion tests were conducted. Before immersion, the samples were cleaned with ethanol and distilled water and then dried. The samples were then sealed with epoxy resin, and only the coated surfaces were exposed to corrosive solutions. Then, the initial weight of each sample was determined using an electronic balance with a precision of 0.1 mg and recorded as G1. Each sample was placed in a beaker with the coated surface facing upwards before adding corrosive solution. The beakers were then placed in a water bath with the temperature maintained at 35 ± 2 °C. Specimens were retrieved at 0.5, 1, 3, 4, 5, 7, and 10 days of immersion. The corrosive solutions were replaced every 12 h. After immersion, the samples were rinsed with deionized water, ultrasonically cleaned (in ethanol, 10 min) to eliminate corrosion products, dried, and weighed. The weight measured after the immersion test was recorded as G2. The mass loss W (mg) due to corrosion was thus calculated using Equation (2) [16]. Thus, the corrosion rate Cr (mm·y^−1^) can be calculated according to the classical weight-loss equation as follows [16]:

(2) $$W=G_{1}-G_{2}$$

(3) $$C_{r}=\frac{87.6W}{\rho At}$$

where W is the mass loss (mg), ρ is the coating density (g·cm^−3^), A is the exposed surface area (cm^2^), and *t* is the immersion time (h). Each test was repeated five times, and the average value was reported.

**Figure 2:**
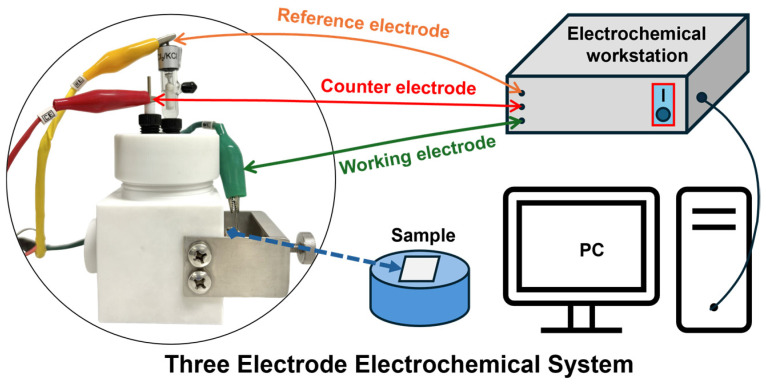
Schematic illustration of the electrochemical corrosion test setup.

The electrochemical corrosion behaviors of WC-10Co-4Cr coatings were investigated using an electrochemical workstation (CS2350H, Wuhan KST Instruments Co., Wuhan, China). The schematic diagram of the electrochemical test setup is shown in Figure 2.

Prior to the experiment, the samples were sealed with epoxy resin, leaving the top surface (coating) exposed to the solution. The sample was then mounted to the three-electrode electrochemical cell with an exposure area of 1 cm^2^. The coating serves as the working electrode (WE), platinum as the counter electrode (CE), and a saturated calomel electrode (SCE) as the reference electrode (RE). They were tested in 0.2 mol/L H_2_SO_4_ and 0.4 mol/L HNO_3_ electrolyte solutions, respectively. All tests were conducted at a room temperature of about 25 °C. Initially, the open-circuit potential (OCP) of the sample was tested after 1 h immersion in the medium. Then, electrochemical impedance spectroscopy (EIS) measurements were performed. The frequency range for EIS was set from 10^5^ Hz to 10^−2^ Hz. Subsequently, the kinetic potentiodynamic polarization (PDP) tests were carried out at a scan rate of 1 mV/s, covering a scan range from 0.5 V to +1.5 V (vs. OCP). After tests, the impedance data were analyzed and fitted using ZSimpWin 3.60 software. Tafel extrapolation was used to determine the corrosion current density (I_corr_) based on the linear region of the polarization curves.

The surface corrosion morphologies of the samples and the chemical composition of corrosion products after immersion and electrochemical corrosion tests were observed using SEM and EDS, respectively. The depth of corrosion pits and surface roughness were measured using an optical profiler (OP, Bruker Contour GT-K 3D, Ettlingen, Germany). X-ray photoelectron spectroscopy (XPS, Thermo Scientific K-Alpha, Waltham, MA, USA) was used to analyze the elemental information in the corrosion products.

## 3. Result and Discussion

### 3.1. Microstructure and Properties of Coatings

**Figure 3:**
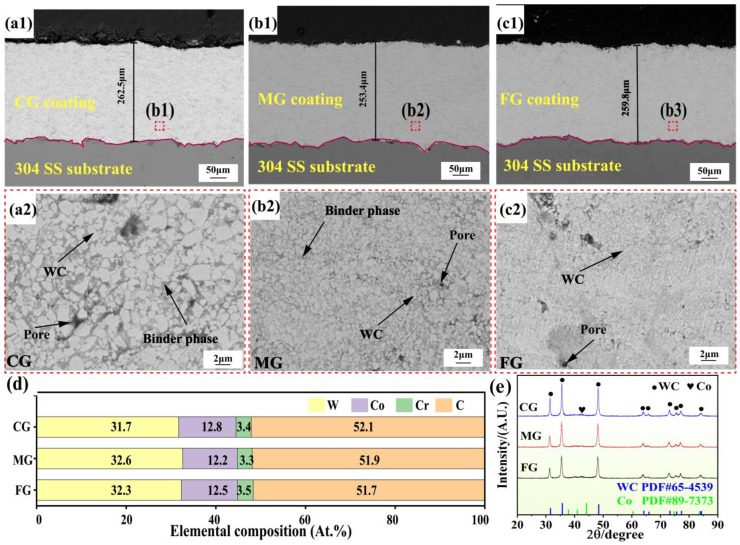
Cross-sectional morphologies of the WC–10Co–4Cr coatings: CG coating (**a1**,**a2**); MG coating (**b1**,**b2**); FG coating (**c1**,**c2**), the EDS analysis (**d**), and XRD analysis (**e**) of the coatings.

The cross-sectional SEM morphologies of CG, MG, and FG WC-10Co-4Cr coatings sprayed on 304 SS substrates are shown in Figure 3(a1–c2). The 304 SS substrate surfaces exhibit a rough and uneven topography due to sandblasting pre-treatment. A distinct interface between the coating and the substrate is evident, indicating that the bonding between them is primarily mechanical in nature, with no detectable elemental diffusion. The three coatings exhibit comparable thicknesses, ranging from 253.4 µm (MG) to 262.5 µm (CG). It is worth noting that although Cr is not presented in the XRD patterns due to the inherent detection limits of the technique, its presence is detected in the EDS analysis. Combining the results of EDS analysis (Figure 3d) and XRD analysis (Figure 3e), it can be concluded that the hexagonal crystal WC hard phase is dominant in the coatings, and the light-colored WC hard phase is uniformly dispersed within the gray CoCr binder phase, with no significant phase segregation (as shown in the higher magnification micrographs in Figure 3(a2–c2)). Among the three coatings, the MG coating comparatively displays a more uniform binder phase distribution with lower pore density compared to the other two coatings. The formation of pores is likely affected by the particle size of WC. Fine WC particles tend to agglomerate and possess insufficient kinetic energy for complete plastic deformation upon impact, leaving unfilled inter-particle voids, while coarse particles suffer from incomplete wetting by the CoCr binder phase during solidification, generating micropores at the WC/CoCr interface. Overall, all three coatings in this study can be considered dense and compact with occasional porosity preferentially located at the WC/CoCr binder phase interface.

**Figure 4:**
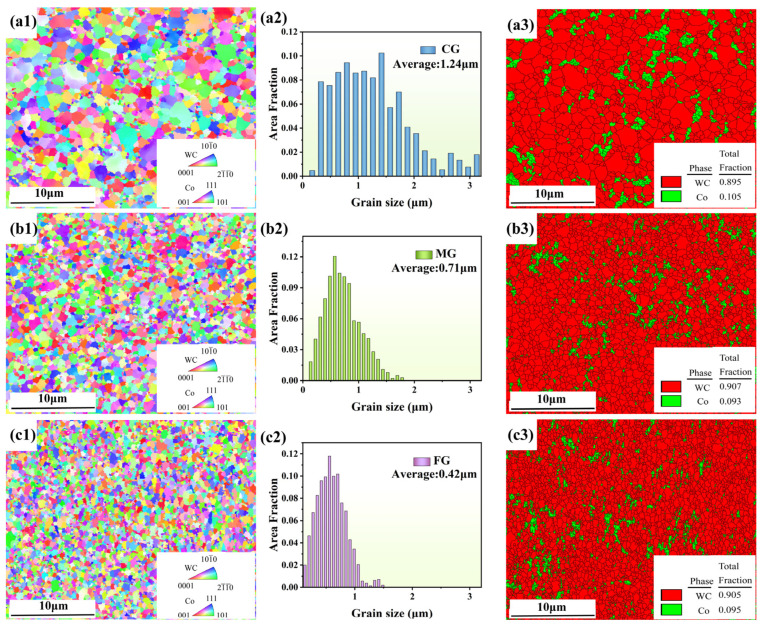
EBSD results of the WC–10Co–4Cr coatings showing crystallographic orientation, grain size distribution and phase composition: CG (**a1**–**a3**); MG (**b1**–**b3**); FG (**c1**–**c3**).

Figure 4 is the EBSD characterization results of the three coatings. The WC grains in all three coatings exhibit random crystallographic orientations. The grain sizes of the coatings are: CG coating displays a grain size range of 0.32 μm to 1.72 μm, with an average grain size of 1.24 μm; MG coating exhibits a grain size range of 0.39 μm to 0.91 μm, with an average grain size of 0.77 μm; and FG coating possesses the finest and most uniform microstructure, with grain sizes ranging from 0.27 μm to 0.84 μm and a mean grain size of 0.42 μm.

In all three coatings, the phase fractions of WC and CoCr binder phase are similar and generally uniformly distributed. This can be confirmed by the small porosity values shown in Table 2, where the coatings exhibit a low porosity variation. The FG coating shows slightly higher porosity and smaller indentation fracture toughness, but a higher bond strength and an obviously higher hardness than the other two coatings. This indicates that the influence of slight porosity differences on strength is limited. Instead, the Hall–Petch mechanism is dominant, i.e., the hardness of materials increases with decreasing grain size, whereas the fracture toughness decreases with decreasing grain size [30]. The results in this study are consistent with the behavior observed in sintered bulk WC–Co cemented carbides [31]. The bonding strengths of the three coatings show little variation and are all higher than 75 MPa, indicating that all coatings possess good bonding performance.

**Figure 5:**
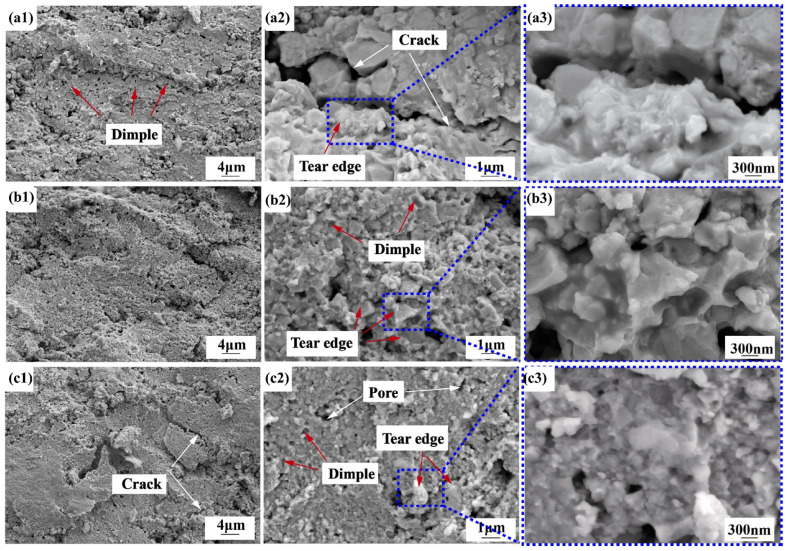
Fracture morphologies of WC–10Co–4Cr coatings: CG (**a1**–**a3**); MG (**b1**–**b3**); FG (**c1**–**c3**).

Figure 5 presents the fracture morphologies of WC-10Co-4Cr coatings with different grain sizes. Low-magnification SEM images in Figure 5(a1–c1) reveal that interfacial debonding of WC particles is the dominant failure mode in all three coatings, resulting in the formation of irregular dimples. The CG coating exhibits the highest number of voids and pits, with large and sparse dimples (Figure 5(a1)) originating from the overall debonding of individual WC particles. The relatively weaker interfacial bonding strength associated with coarse grains facilitates rapid crack propagation along the interface, while the low grain boundary density promotes relatively straight crack trajectories, collectively contributing to the large crack observed in this coating. In contrast, the FG coating (Figure 5(c1)) features fine, densely distributed dimples formed by the collective debonding of multiple fine WC particles. The high-grain boundary density compels cracks to deflect or transgranularly propagate, increasing crack path tortuosity and imparting greater resistance to crack propagation. Although grain refinement enhances the plastic deformation capacity of the binder phase, it simultaneously promotes a more heterogeneous binder distribution, with locally depleted regions susceptible to rapid microcrack coalescence—a factor that partially offsets the toughness improvement. The fracture behavior of the MG coating (Figure 5(b1)) is intermediate between that of the CG and FG coatings, characterized by uniformly distributed fine voids in conjunction with a reduced number of shorter cracks.

### 3.2. Immersion Corrosion

**Figure 6:**
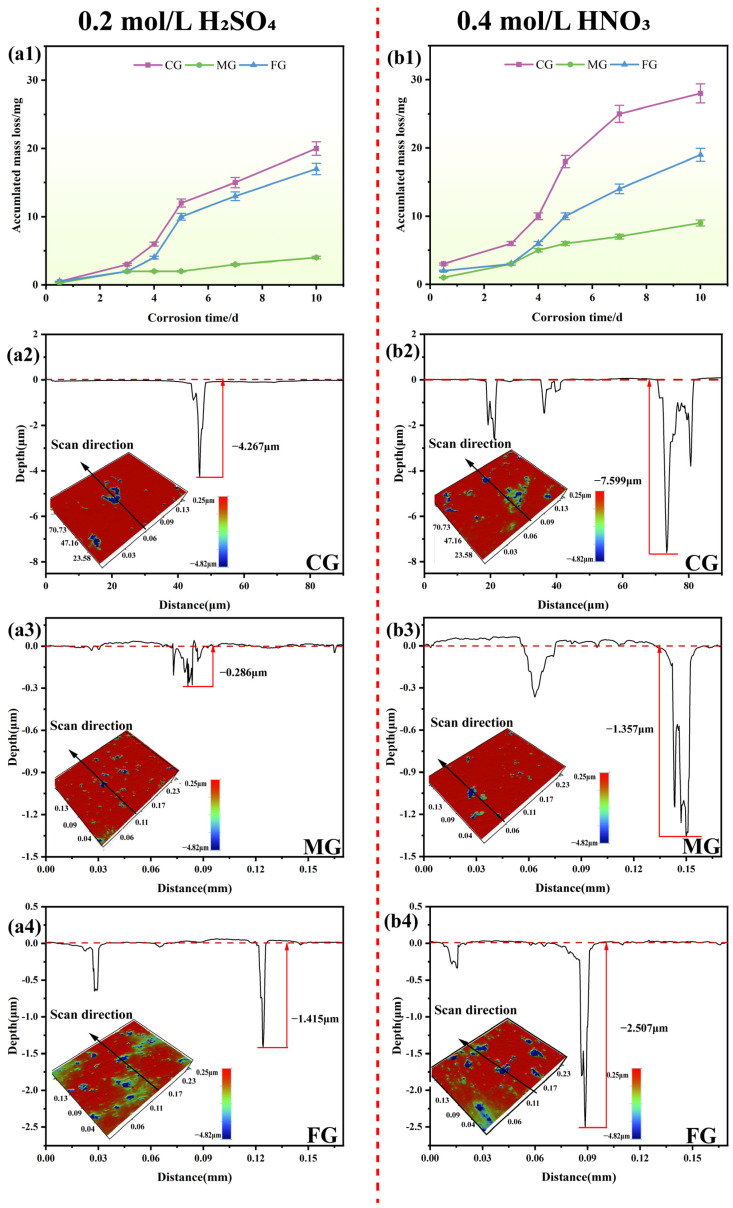
Mass loss of WC–10Co–4Cr coatings in (**a1**) 0.2 mol/L H_2_SO_4_ and (**b1**) 0.4 mol/L HNO_3_ solutions, and the three-dimensional surface morphologies after 10 days of immersion in these solutions: CG coating (**a2**,**b2**); MG coating (**a3**,**b3**); FG coating (**a4**,**b4**).

The average mass losses for CG, MG, and FG coatings after 10 days of immersion in 0.2 mol/L H_2_SO_4_ are 20 mg/cm^2^, 4 mg/cm^2^, and 17 mg/cm^2^, respectively. The corresponding values in 0.4 mol/L HNO_3_ are 28 mg/cm^2^, 9 mg/cm^2^, and 19 mg/cm^2^. The calculated corrosion rates for CG, MG, and FG coatings in 0.2 mol/L H_2_SO_4_ are 0.52 mm·year^−1^, 0.10 mm·year^−1^, and 0.44 mm·year^−1^, respectively. The corresponding values in 0.4 mol/L HNO_3_ are 0.73 mm·year^−1^, 0.23 mm·year^−1^, and 0.50 mm·year^−1^. Figure 6(a1) and Figure 6(b1) further show the relationship between accumulative mass loss and immersion time for the coatings in 0.2 mol/L H_2_SO_4_ and 0.4 mol/L HNO_3_ solutions, respectively. All three coatings exhibit higher mass losses and corrosion rates in the 0.4 mol/L HNO_3_ solution compared to those in the 0.2 mol/L H_2_SO_4_ solution. This difference is attributed to the strong oxidizing nature of HNO_3_, which promotes rapid oxidation of the metallic binder phase, forming soluble metal ions and thereby accelerating the structural degradation of the coating. Notably, in the 0.4 mol/L HNO_3_ medium, oxide corrosion products were visible upon recovery from the immersion solution, whereas no such corrosion products were observed on the samples retrieved from the 0.2 mol/L H_2_SO_4_ solution. In both corrosive acids, the corrosion rate shows a power law relationship with the corrosion time, which is consistent with the classic corrosion model. Under identical corrosion durations, the mass loss of the CG coating is the largest, while the MG coating is the lowest in both media, indicating a superior corrosion resistance of the MG coating. For FG coating with the finest microstructure, its lower corrosion resistance is likely due to the slightly higher porosity, which serves as a preferential pathway for corrosion medium penetration.

The three-dimensional surface morphologies of corroded coatings measured by optical profilometry after 10 days of immersion are presented in Figure 6(a2–b4). The results confirm that the CG coating exhibits the poorest corrosion resistance in both solutions, presenting the greatest pit depth (4.267 μm in 0.2 mol/L H_2_SO_4_ and 7.599 μm in 0.4 mol/L HNO_3_). In contrast, the MG coating demonstrates the best performance, suffering the least damage (0.286 μm in 0.2 mol/L H_2_SO_4_ and 1.357 μm in 0.4 mol/L HNO_3_). The FG coating displays an intermediate level of damage (1.415 μm in 0.2 mol/L H_2_SO_4_ and 2.507 μm in 0.4 mol/L HNO_3_). These findings indicate that the corrosion resistance of the HVAF sprayed WC–10Co–4Cr coatings was synergistically influenced by WC grain size and microstructural porosity. The CG coating, with its coarse grains and sparse grain boundaries, tends to concentrate the corrosion current on a few sites, accelerating localized dissolution. Once these boundaries are breached, entire large grains detach, forming deep pits that propagate and rapidly roughen the surface, markedly diminishing corrosion resistance. Conversely, the FG coating, despite its fine grains and short grain-boundary migration distances, fails to effectively eliminate porosity, allowing the corrosive medium to readily penetrate the coating surface.

**Figure 7:**
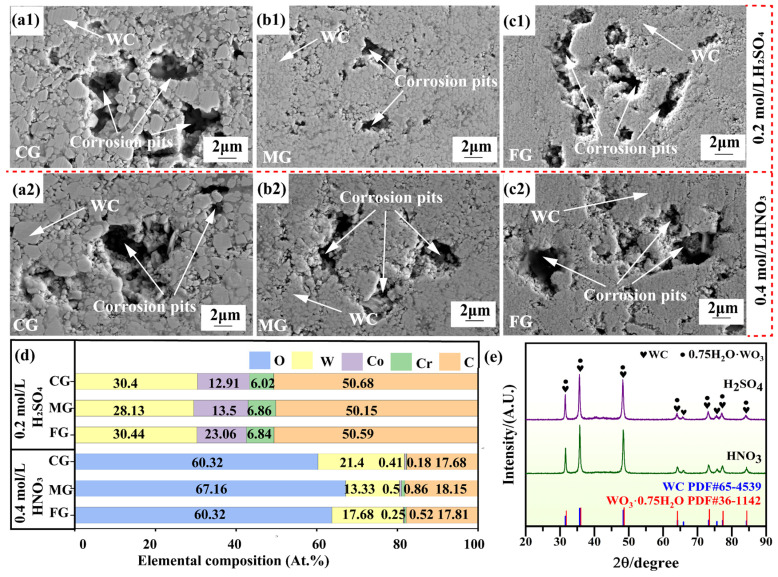
Surface morphologies of corroded coatings after immersion in 0.2 mol/L H_2_SO_4_ (CG (**a1**), MG (**b1**), and FG (**c1**) coatings, respectively) and 0.4 mol/L HNO_3_ solutions (CG (**a2**), MG (**b2**), and FG (**c2**) coatings, respectively), (**d**) the EDS analysis of the corroded surfaces, and (**e**) XRD pattern of the corrosion products.

Figure 7(a1–c2) shows the surface morphologies of the corroded coatings. It is further revealed that the pit density of the MG coating is lower than that of the CG and FG coatings, consistent with the smallest accumulative mass loss and lowest pit depths presented above, thereby corroborating the superior corrosion resistance of the MG coating. The corrosion damage initiated preferentially at the WC/CoCr interphases, forming typical corrosion trenches. This phenomenon originates from the electrochemical micro-galvanic cell formed in the acidic medium, where the WC phase acts as the cathode and the CoCr binder phase undergoes preferential dissolution as the anode. The electrochemical reaction ultimately leads to WC particle debonding and pit formation.

The EDS elemental analysis of the corroded regions (as shown in Figure 7d) shows that a large amount of O is present on the sample surfaces immersed in the 0.4 mol/L HNO_3_ solution, whereas no O is detected for samples immersed in the 0.2 mol/L H_2_SO_4_ solution. The atomic percentage of W and C elements increases slightly (1.4%), and the fraction of Cr doubles compared to the elemental composition of samples before immersion (Figure 3d), confirming the chemical inertness of the WC hard phase and the corrosion resistance property of Cr. The Co element loss in the binder phase exceeded 2%, verifying the preferential dissolution of the metallic binder phase. According to the XRD analysis shown in Figure 7e, the oxide formed in the HNO_3_ environment is WO_3_·0.75H_2_O.

**Figure 8:**
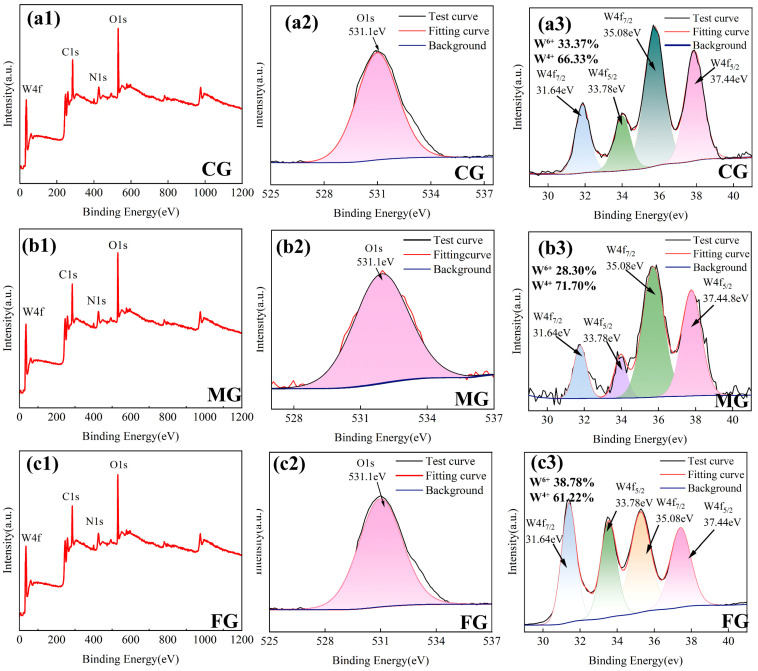
XPS spectra of the coatings after immersion in 0.4 mol/L HNO_3_ solution: CG (**a1**–**a3**); MG (**b1**–**b3**); FG (**c1**–**c3**).

To further define the presence of oxide formed in the 0.4 mol/L HNO_3_ solution, XPS analysis was applied, and the results are shown in Figure 8. The W 4f spectrum exhibited characteristic double peaks at 35.08 eV (W 4f_7_/_2_) and 37.44 eV (W 4f_5_/_2_), confirming the presence of the W^6+^ oxidation state. Additionally, the strong peak at 531.1 eV in the O 1s spectrum corresponds to the W-O chemical bond. Collectively, these results suggest that the hard phase WC was oxidized to WO_3_·0.75H_2_O during the corrosion process. The related corrosion reactions are as follows [19,30]:

(4) $$WC+2{HNO}_{3}=WO_{3}+H_{2}↑+\;{CO}_{2}↑+\;NO↑$$

(5) $$WO_{3}+0.75H_{2}=WO_{3}\cdot 0.75H_{2}O$$

By comparing the W^6+^ and W4^+^ proportion shown in Figure 8(a3–c3), the proportion of W^6+^ (representing the content of WO_3_·0.75H_2_O oxide) is the smallest for MG (28.3% compared to 33.37% for CG and 38.78% for FG). This is another piece of evidence supporting that the MG coating has the highest corrosion resistance among the three coatings.

### 3.3. Electrochemical Corrosion

#### 3.3.1. OCP and Potentiodynamic Polarization Tests

**Figure 9:**
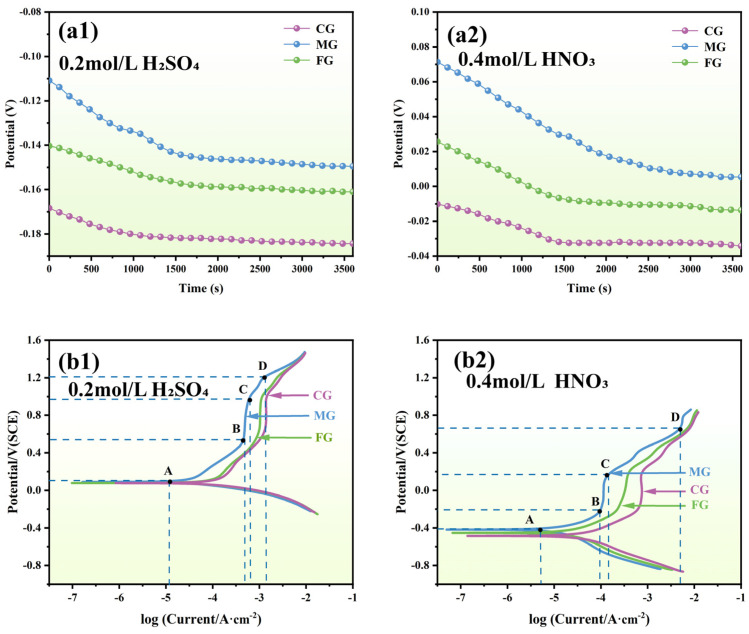
OCP curves (**a1**,**a2**) and potentiodynamic polarization curves (**b1**,**b2**) of CG, MG, and FG coatings after 1 h immersion in 0.2 mol/L H_2_SO_4_ and 0.4 mol/L HNO_3_ solutions.

Figure 9(a1) and Figure 9(a2) present the OCP curves of the three types of coatings (CG, MG, and FG) after 1 h of immersion in 0.2 mol/L H_2_SO_4_ and 0.4 mol/L HNO_3_, respectively. The experimental results indicate that the OCP values of all three coatings gradually decreased and stabilized within 25 min (with fluctuations < ±5 mV), suggesting the formation of a stable corrosion product layer on the coating surface in the acidic medium, which may effectively suppress the initiation of localized pitting. In HNO_3_, the stable potential of the MG coating was −0.14 V, which is 40 mV and 20 mV more positive than that of CG (−0.18 V) and FG (−0.16 V), respectively. In H_2_SO_4_, the stable potential of MG (0.005 V) was also significantly higher than that of CG (−0.033 V) and FG (−0.013 V). According to the principles of corrosion thermodynamics, a higher stable OCP indicates lower electrochemical activity and a stronger passivation ability of the material [10]. This data further confirms that MG exhibits the best corrosion resistance in both acidic environments, which is highly consistent with the previous weight loss test and micro-morphology analysis results.

Figure 9(b1,b2) present the polarization curves of three types of coatings (CG, MG, and FG) in 0.2 mol/L H_2_SO_4_ and 0.4 mol/L HNO_3_, which can be divided into four typical stages: (1) Active dissolution region (A,B): In HNO_3_ solution, when the potential is below −0.13 V, and in H_2_SO_4_ solution, when the potential is below 0.12 V, the Co binder phase preferentially undergoes anodic dissolution (Co → Co^2+^ + 2e^−^) [19], resulting in an exponential increase in corrosion current density with the positive shift in potential. (2) Stable passivation region (B,C): As the corrosion potential increases, the increasing rate of corrosion current density decreases, reaching a stable region. The oxidation reaction of Cr (Cr + 2H_2_O → CrO_2_^−^ + 4H^+^ + 3e^−^) [19], which dominates and forms a Cr-based passive film on the surface, inhibits further electrochemical corrosion. The corrosion current density stabilizes in the range of 0.46–0.59 mA/cm^2^ in HNO_3_ and 0.1–0.14 mA/cm^2^ in H_2_SO_4_, respectively. (3) Overpassivation region (C,D): As the potential continues to increase, the passivated state of the coating is disrupted (CrO_2_^−^ +2H_2_O → CrO_4_^2−^ + 4H^+^ + 2e^−^) [19], and the corrosion is accelerated, leading to a near-linear increase in corrosion current density. The current density in HNO_3_ solution sharply rises to 1.35 mA/cm^2^, while it increases to 4.9 mA/cm^2^ in H_2_SO_4_ solution. (4) Pseudo passivation region (after point D) (Figure 9(b1)): As the corrosion potential increases, the current density continues to rise but at a progressively slower rate. This is primarily due to the accumulation of the corrosion product WO_3_ at the surface, which hinders diffusion of the corrosive species into the deeper coating and results in a phenomenon similar to passivation.

The Tafel fitting data (Table 3) confirm that MG exhibits the best electrochemical performance. In HNO_3_ solution, the corrosion potential of MG (−0.360 V) is 18 mV and 17 mV more positive than that of coarse-grained CG (−0.348 V) and ultrafine-grained FG (−0.347 V), respectively. Also, its corrosion current density (0.03 mA/cm^2^) is reduced by 70% compared to CG (0.10 mA/cm^2^) and by 67% compared to FG (0.09 mA/cm^2^). In the H_2_SO_4_ environment, this advantage is more pronounced. The corrosion potential of MG (−0.081 V) is 65 mV more positive than that of CG (−0.146 V), and its corrosion current density (0.01 mA/cm^2^) is reduced by 66% compared to CG and by 50% compared to FG. Overall, the electrochemical activity and corrosion sensitivity of the three coatings follow the sequence of MG > FG > CG.

#### 3.3.2. Electrochemical Impedance Spectroscopy

**Figure 10:**
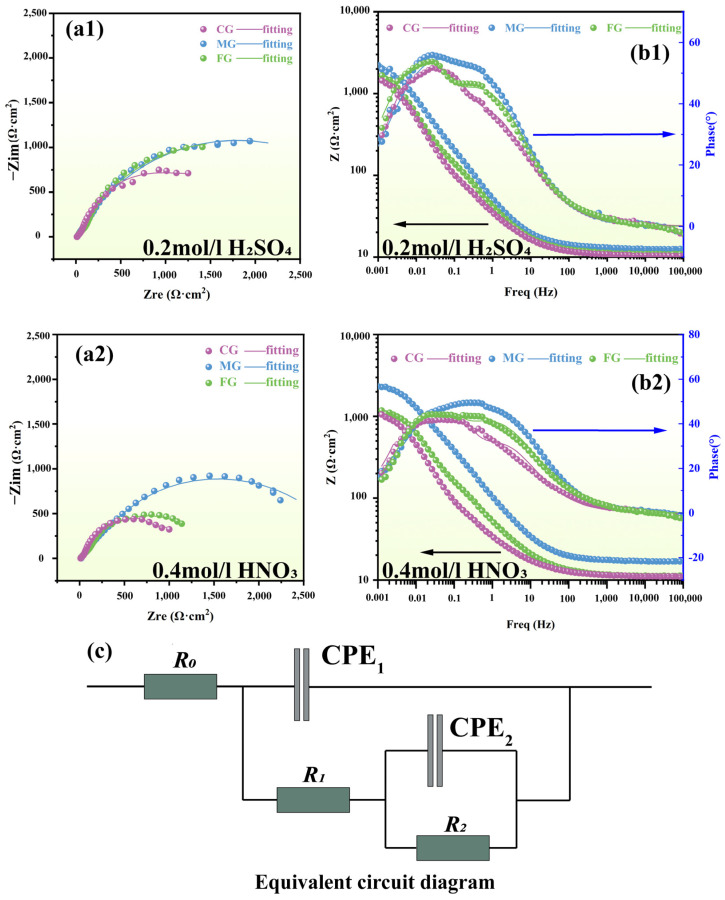
Nyquist and Bode plots of the three coatings in 0.2 mol/L H_2_SO_4_ (**a1**,**b1**) and 0.4 mol/L HNO_3_ solutions (**a2**,**b2**) and the equivalent circuit model (**c**).

From the Nyquist and Bode plots of the three different coatings tested in 0.2 mol/L H_2_SO_4_ and 0.4 mol/L HNO_3_ solutions shown in Figure 10, all coatings exhibited similar impedance behavior in both media, i.e., an indistinct capacitive arc appeared in the high-frequency region, accompanied by a larger capacitive arc in the low-frequency region. The Bode phase plots in Figure 10(b1,b2) reveal double time constants, with the second phase peak in the high-frequency region being partially obscured. The first high-frequency arc, corresponding to the pre-dissolution/adsorption initiation of cobalt within the electric double layer, reflects the process in which cobalt atoms in the binder phase lose electrons and form cobalt ions in the double layer; the second low-frequency arc represents the charge-transfer resistance (R_ct_), and an increase in its radius signifies enhanced corrosion resistance [32]. MG exhibited the highest R_ct_, followed by FG and then CG. This trend is consistent with the previous analysis of polarization curves, corrosion current density, and corrosion potential. The characteristics of the outer layer are reflected at high frequencies, while those of the inner layer are reflected at mid-frequencies, and the low-frequency range reflects the intersection of the substrate and coating [33]. A high-frequency phase angle dropping below 20° indicates a gradual decline in the protective capability of the coating. At mid-frequencies, the phase angles of all coatings rapidly decrease, indicating coating penetration and the onset of corrosion degradation. During this stage, the decline in phase angle for the MG coating is relatively slow, likely due to the deposition of corrosion degradation products at coating defect sites. Overall, the MG coating demonstrates the best corrosion resistance.

An equivalent circuit model shown in Figure 10c was used to fit the impedance data, which has been previously applied in studies of plasma-sprayed WC-Co coatings [34]. The circuit consists of the solution resistance ($R_{0}$), two resistive elements ($R_{1}$ and $R_{2}$) associated with interfacial electrochemical processes, and two constant phase elements (CPE_1_ and CPE_2_) introduced to replace ideal capacitors in order to account for surface and interface inhomogeneity. The impedance of the CPE is defined as [6]

(6) $$Z_{CPE}=1/Q^{0}{\left(j\omega \right)}^{\alpha }$$

Calculations indicate that the equivalent capacitance value of the MG coating is lower than that of the other two coatings. A large amount of charge tends to accumulate preferentially along the WC/CoCr interface. When the charge concentration reaches a critical value, it will trigger the dissolution of the binder phase, leading to a decrease in the charge transfer resistance of the coating. In comparison, the charges in the MG coating are more uniformly distributed on the surface of the binder phase, thus avoiding local charge accumulation. This conclusion can be verified by comparing the charge transfer resistance $R_{1}$. The impedance expression of the equivalent circuit is [6]

(7) $$Z=R_{0}+\frac{1}{\frac{1}{\frac{1}{\frac{1}{R_{2}}+Q_{2}^{0}{\left(jw\right)}^{\alpha 2}}+R_{1}}+Q_{1}^{0}{\left(jw\right)}^{\alpha 1}}$$

Here, $Q^{0}$ is the CPE coefficient, α is the CPE exponent (0 ≤ α ≤ 1), j is the imaginary unit, and ω is the angular frequency. When ω → 0, the total polarization resistance ($R_{p}$) satisfies $R_{p}$ = $R_{1}$ + $R_{2}$. The fitting data of the EIS for coatings tested in the 0.2 mol/L H_2_SO_4_ and 0.4 mol/L HNO_3_ solutions are summarized in Table 4 and Table 5, respectively. The calculations reveal that the $R_{p}$ values of MG are always higher than those of CG and FG under the same conditions, indicating its lower electron mobility and conductivity. Since the material’s polarization resistance is inversely proportional to the corrosion rate, the EIS results further demonstrate that the MG coating exhibits the best corrosion resistance. This explains why the corrosion current density and corrosion rate of MG are lower than those of CG and FG. The equivalent circuit fitting with (χ^2^) values in the range of 10^−2^ to 10^−5^ indicates good fitting quality [35].

#### 3.3.3. Corrosion Morphology and Composition Analysis

**Figure 11:**
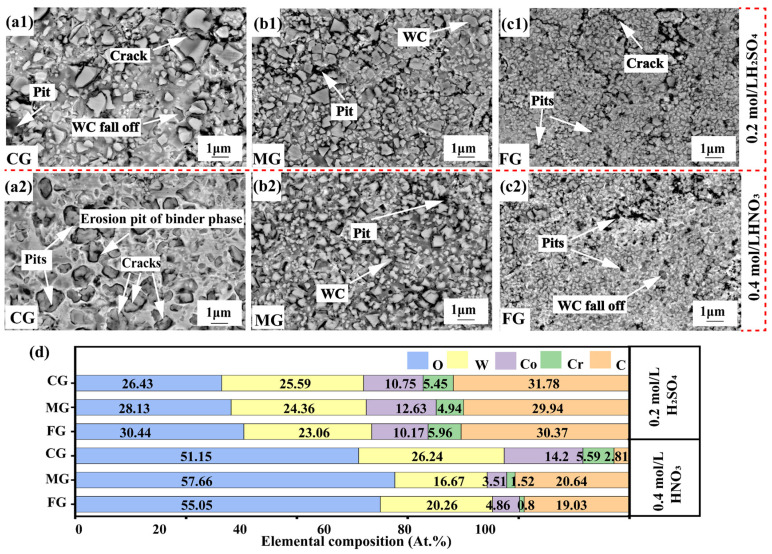
Corrosion morphologies of CG (**a1**,**a2**), MG (**b1**,**b2**), and FG (**c1**,**c2**) coatings in 0.2 mol/L H_2_SO_4_ solution and 0.4 mol/L HNO_3_ solutions, and the EDS analysis results (**d**).

Figure 11(a1–c2) illustrate the post-corrosion surface morphologies of the three WC-10Co-4Cr coatings. Most of the WC hard phases are surrounded by a dark halo, which originates from the preferential dissolution of the CoCr binder phase at the immediate periphery of the WC particles. This phenomenon is triggered by the galvanic corrosion between WC and the CoCr binder phase under an applied voltage/current: Co undergoes selective dissolution due to its lower corrosion potential, especially at the WC/CoCr interface. The continuous dissolution of the binder phase ultimately leads to the detachment of WC particles, forming characteristic pits.

Research indicates that the addition of Cr significantly enhances corrosion resistance through a threefold synergistic mechanism, including ① forming a denser Co-Cr binder phase, ② generating a protective Cr_2_O_3_ oxide film in situ during the corrosion process, and ③ leveraging Cr’s own passivation ability to protect the binder phase from dissolution. This is supported by the EDS analysis as shown in Figure 11d, where the increased O content and decreased Co content within the corroded areas indicate electrochemical dissolution of Co [36].

Grain size impacts the coating defect density and thus has a decisive influence on the degree of corrosion. MG exhibits a few shallow surface pits and microcracks. CG shows a high-density network of pits, and FG displays extensive microcrack propagation. This corrosion pattern is consistent with the Tafel polarization and Nyquist analysis results. The superior corrosion resistance of MG benefits from the fewer pores and compact microstructure during manufacturing. CG forms more pores due to the larger intergranular spacing, while the FG coating develops microcracks influenced by residual stress. These structural defects provide preferential pathways for the penetration of corrosive media. Within the acidic media, the leaching of Co^2+^ and Cr^3+^ destabilizes the passive film structure. When the amount of binder phase dissolution exceeds a critical value, it triggers the detachment of WC particles, forming characteristic pit structures. The pits in Figure 7 are formed when the binder phase dissolves to a certain extent and can no longer encapsulate the WC particles, eventually causing the detachment of these particles.

#### 3.3.4. Mechanism of Electrochemical Corrosion

**Figure 12:**
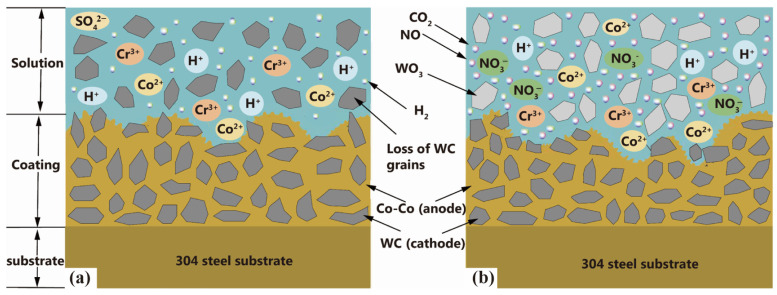
Schematic diagrams showing the corrosion mechanisms of WC-10Co-4Cr coatings in (**a**) 0.2 mol/L H_2_SO_4_ and (**b**) 0.4 mol/L HNO_3_.

The above results indicate that the MG coating exhibits the best corrosion resistance in both 0.2 mol/L H_2_SO_4_ and 0.4 mol/L HNO_3_ solutions, followed by the FG coating, with the CG coating showing the poorest performance. The corrosion resistance of the coatings is significantly correlated with microstructural defects (pores, cracks, and binder phase composition and proportion), and the different oxidizing nature of the acids results in different corrosion mechanisms and corrosion products. Figure 12 presents the schematic view of the corrosion models of the three coatings in the two acidic environments. The CG coating, with its large WC grain size, has wider grain boundaries and more pores, allowing the corrosive solution to penetrate rapidly and accelerate the corrosion in both acid solutions. The FG coating, with the smallest grain size and a relatively high porosity, provides rapid penetration pathways for H^+^ and SO_4_^2−^ through surface pores and interlayer cracks in 0.2 mol/L H_2_SO_4_, accelerating the anodic dissolution reaction of Co in the binder phase.

Since H_2_SO_4_ has a relatively weak oxidation ability towards WC, the initial corrosion is primarily characterized by the dissolution of the Co phase. The fine WC particles, having insufficient pinning depth, are prone to detachment from the substrate after the Co phase is corroded. This leads to surface roughening and the exposure of new active areas, further promoting the hydrogen evolution reaction [19,30].

(8) $$2H^{+}+2e^{-}\to H_{2}↑$$

High porosity and interconnected pores allow the corrosive medium to penetrate rapidly into the coating, establishing an autocatalytic cycle that ultimately triggers synergistic uniform corrosion and particle detachment. In CG and FG coatings, coarse pores and non-uniform WC distribution create local WC-rich cathodes and adjacent CoCr binder anodes; the resulting potential difference drives micro-galvanic cells in which the Co anode is forced to supply more electrons and dissolves preferentially, so the more WC particles agglomerate, the more severe the corrosion. By contrast, the MG coating’s low porosity and uniformly dispersed WC produce a relatively homogeneous potential field, markedly weakening micro-galvanic effects and yielding only mild attack. In addition, the in situ Cr_2_O_3_ passive film, though only marginally stable in nitric acid, still partly suppresses anodic dissolution and further reduces the corrosion rate. In the HNO_3_ environment, the strong oxidizing nature of HNO_3_ fundamentally alters the corrosion mechanism. Nitrate ions (NO_3_^−^) directly participate in the cathodic reduction reaction (Equation (7)), accelerating the oxidative dissolution of Co and Cr through the following equations [19,30]:

(9) $$\mathrm{Cathode}:\;{NO_{3}}^{-}+4H^{+}+3e^{-}\to NO↑+\;2H_{2}O$$

(10) $$\mathrm{Anode}:\;Co\to {Co}^{3+}+3e^{-};\;Cr\to {Cr}^{3+}+3e^{-}$$

For the FG coating, the high porosity allows HNO_3_ to penetrate rapidly. The detached WC particles are instantaneously oxidized (Equation (9)) [19,30], forming porous WO_3_ and gaseous products, which trigger layer-by-layer spalling and pitting. The interlayer cracks serve as preferential pathways for the longitudinal diffusion of nitric acid, leading to delamination.

(11) $$WC+10HNO_{3}\to WO_{3}+CO_{2}↑+\;10NO_{2}↑+\;5H_{2}O$$

However, the strong oxidizing nature of HNO_3_ diminishes this advantage. Once the corrosive medium penetrates through local defects, the galvanic corrosion at the WC/CoCr interface is intensified. Meanwhile, the oxidation of WC to WO_3_ weakens the mechanical support, ultimately leading to structural collapse—a phenomenon particularly pronounced in layered coatings, where corrosion propagates along interlayer weak zones to form a “sandwich-like” corrosion morphology.

## 4. Conclusions

The main conclusions of this study are summarized as follows:

(1) WC grain size influences the microstructure and corrosion resistance of HVAF-sprayed WC-10Co-4Cr coatings. Among the three coatings studied, the MG coating (average WC grain size of 0.77 μm) achieves the highest corrosion resistance.
(2) The corrosion resistance of HVAF-sprayed WC-10Co-4Cr coatings is synergistically controlled by WC grain size and microstructural defect density. In both 0.2 mol/L H_2_SO_4_ and 0.4 mol/L HNO_3_ media, corrosion initiates through preferential anodic dissolution of Co in the CoCr binder phase driven by micro-galvanic coupling with the WC hard phase, followed by progressive WC particle detachment and pit formation. The CG coating exhibits the worst corrosion resistance owing to its wide grain boundaries and high porosity, while the FG coating is similarly compromised by slightly higher porosity and residual stress-induced microcrack networks that facilitate electrolyte penetration.
(3) Electrochemical measurements confirm that the MG coating exhibits the most noble corrosion potential, the lowest corrosion current density, and the highest total polarization resistance. These features are attributable to its compact microstructure and uniform binder phase distribution, which suppress micro-galvanic activity and promote a more homogeneous passive film. EIS analysis further reveals that the MG coating has the lowest equivalent capacitance and the highest charge transfer resistance, indicating more effective inhibition of charge-carrier transport across the coating–electrolyte interface.
(4) The two acidic environments impose distinct corrosion mechanisms. In 0.2 mol/L H_2_SO_4_, corrosion proceeds primarily by selective Co dissolution and H_2_ evolution, with limited oxidation of the WC phase. In 0.4 mol/L HNO_3_, the strong oxidizing nature of NO_3_^−^ accelerates both binder dissolution and direct WC oxidation, generating WO_3_·0.75H_2_O corrosion product. This oxide layer partially retards further diffusion of corrosive species but also weakens mechanical support, eventually leading to WC particle detachment and pitting.

The results of this study provide a valuable reference for HVAF-sprayed WC-10Co-4Cr coatings used in typical industrial acidic environments. To better understand the coating performance under actual service conditions, further investigations should focus on the dynamic corrosion or wear–corrosion synergistic behavior of this coating material. Moreover, long-term immersion or field service evaluation experiments are necessary to validate the coating’s long-term durability in real-world environments.

## Figures and Tables

**Table 1:** Parameters of the HVAF spraying process.

Atmosphere /PSI	Fuel Pressure /PSI	Nitrogen Flow Rate /Slpm	Chamber Pressure/PSI	Substrate Temperature °C	Powder Feed Rate g/min	Spray Distance mm
95	83	23	66	100~150	100	150

**Table 2:** Mechanical properties of WC-10Co-4Cr coatings with different WC grain sizes.

Coating	Porosity (%)	Bonding Strength (MPa)	Hardness (*HV*_30_)	Indentation Fracture Toughness (*K_IC_*, MPa·m^1/2^)
CG	0.9 ± 0.1	78 ± 1.4	1153 ± 8.8	6.9 ± 0.2
MG	0.9 ± 0.1	79 ± 2.0	1195 ± 7.8	5.9 ± 0.2
FG	1.1 ± 0.1	80 ± 2.0	1239 ± 9.6	5.2 ± 0.2

**Table 3:** Fitting parameters of potentiodynamic polarization curves.

Solution	Specimens	*E*_corr_ (V)	χ^2^ (1 × 10^−2^)	$\beta_{a}$ (mV·dec^−1^)	$-\beta_{c}$ (mV·dec^−1^)	*i*_corr_ (mA·cm^2^)
H_2_SO_4_	CG	0.15 ± 0.008	2.27 ± 0.100	105.10 ± 4.730	251.01 ± 11.300	0.03 ± 0.004
	MG	0.08 ± 0.005	1.47 ± 0.007	103.43 ± 4.650	305.62 ± 13.750	0.01 ± 0.002
	FG	0.11 ± 0.007	2.00 ± 0.009	110.76 ± 4.900	299.91 ± 13.500	0.02 ± 0.003
HNO_3_	CG	−0.35 ± 0.012	5.22 ± 0.240	581.50 ± 26.170	74.60 ± 3.360	0.10 ± 0.010
	MG	−0.33 ± 0.009	3.36 ± 0.150	559.80 ± 25.190	5.90 ± 0.270	0.03 ± 0.004
	FG	−0.35 ± 0.011	3.64 ± 0.160	416.40 ± 18.740	−28.00 ± 1.260	0.09 ± 0.009

**Table 4:** Fitting data of EIS for coatings tested in the 0.2 mol/L H_2_SO_4_ environment.

Parameters	CG	MG	FG
$R_{0}$ (Ω·cm^2^)	11.40 ± 0.520	16.81 ± 0.760	11.38 ± 0.051
$Q_{1}^{0}$ (×10^−3^ Ω^−1^ cm^−2^ S^n^)	7.63 ± 0.340	3.1 ± 0.140	5.83 ± 0.026
α1	0.68 ± 0.028	0.65 ± 0.026	0.64 ± 0.025
$R_{1}$ (kΩ·cm^2^)	0.07 ± 0.003	1.78 ± 0.081	0.282 ± 0.013
$Q_{2}^{0}$ (×10^−3^ Ω^−1^ cm^−2^ S^n^)	0.17 ± 0.003	4.72 ± 0.212	7.10 ± 0.320
α2	0.95 ± 0.030	0.99 ± 0.010	0.99 ± 0.010
$R_{2}$ (kΩ·cm^2^)	1.24 ± 0.056	1.34 ± 0.061	1.26 ± 0.057
$R_{p}$ (kΩ·cm^2^)	1.26 ± 0.057	3.14 ± 0.142	1.56 ± 0.070
χ^2^ (1 × 10^−2^)	0.87 ± 0.039	0.33 ± 0.015	0.78 ± 0.035

**Table 5:** Fitting data of EIS for coatings tested in the 0.4 mol/L HNO_3_ environment.

Parameters	CG	MG	FG
$R_{0}$ (Ω·cm^2^)	10.26 ± 0.460	12.80 ± 0.580	11.08 ± 0.500
$Q_{1}^{0}$ (×10^−3^ Ω^−1^ cm^−2^ S^n^)	17.81 ± 0.800	6.26 ± 0.280	7.91 ± 0.360
α_1_	0.98 ± 0.004	0.68 ± 0.028	0.66 ± 0.027
$R_{1}$ (kΩ·cm^2^)	0.41 ± 0.018	1.53 ± 0.069	1.09 ± 0.049
$Q_{2}^{0}$ (×10^−3^ Ω^−1^ cm^−2^ S^n^)	33.61 ± 1.510	2.71 ± 0.122	8.06 ± 0.363
α_2_	0.99 ± 0.010	0.99 ± 0.010	0.99 ± 0.010
$R_{2}$ (kΩ·cm^2^)	0.87 ± 0.039	3.04 ± 0.137	2.38 ± 0.107
$R_{p}$ (kΩ·cm^2^)	1.28 ± 0.058	4.57 ± 0.206	3.47 ± 0.156
χ^2^ (1 × 10^−2^)	0.93 ± 0.042	0.61 ± 0.027	0.61 ± 0.027

## Data Availability

The original contributions presented in this study are included in the article. Further inquiries can be directed to the corresponding authors.

## Abbreviations

The following abbreviations are used in this manuscript:

HVAF	High-velocity air-fuel
HVOF	High-velocity oxygen-fuel
